# Laennec’s approach for laparoscopic anatomical hemihepatectomy

**DOI:** 10.1186/s12957-021-02404-1

**Published:** 2021-10-06

**Authors:** Wei Hu, Gongming Zhang, Meng Chen, Chengcheng Zhong, Mingxu Li, Xitai Sun, Kai Li, Zhong Wang

**Affiliations:** 1 Department of Hepatobiliary Surgery, The First Affiliated Hospital of Kangda College of Nanjing Medical University, Nanjing, 222001 Jiangsu China; 2 Department of Hepatobiliary Surgery, Affiliated Lianyungang Hospital of Xuzhou Medical University, Lianyungang, 222001 Jiangsu China; 3 Department of Pathology, The First Affiliated Hospital of Kangda College of Nanjing Medical University, Nanjing, 222001 Jiangsu China; 4 Department of Hepatobiliary Surgery, Nanjing Drum Tower Hospital, The Affiliated Hospital of Nanjing University Medical School, Nanjing, 210093 Jiangsu China

**Keywords:** Laparoscopy, Hemihepatectomy, Laennec’s capsule, Surgical approach

## Abstract

**Background:**

Laennec’s capsule has been found for about 200 years. However, laparoscopic anatomical right and left hemihepatectomy (LARH and LALH) using Laennec’s approach are rarely reported.

**Methods:**

We retrospectively analyzed the technical details and the surgical outcomes of 15 patients who underwent LAH via Laennec’s approach between May 2017 and July 2020. The operation time, intraoperative blood loss, postoperative complications, and hospital stay were recorded and analyzed.

**Results:**

Four of 15 patients were diagnosed with hepatic hemangioma, 2 had hepatolithiasis, and 9 patients had primary liver cancer. During the surgery, Laennec’s approach was used for LAH without conversion to open surgery. Four patients were treated with LARH, and 11 patients were cured with LALH. The mean age of the patients was 62.1 ± 6.5 years, and four were male. The mean operative time, blood loss, and length of the postoperative hospital stay were 193 ± 49 min, 247 ± 120 mL, and 8.7 ± 2.0 days, respectively. There was no incidence of postoperative bile leakage and bleeding. No mortality occurred. We also demonstrated that Laennec’s capsule does exist around the peripheral hepatic veins with histological confirmation.

**Conclusions:**

Laennec’s approach is safe and feasible for LAH. Precise isolation of Laennec’s approach based on Laennec’s capsule helps to standardize the surgical techniques for laparoscopic anatomical hepatectomy.

## Introduction

Laparoscopic liver surgery, a widely accepted standard surgical practice for the management of liver neoplasm, has evolved over the past two decades, and the procedure has expanded from initial local hepatectomy to anatomical hepatectomy. Laparoscopic hepatic resection has attained an equivalent status of safety and efficacy as conventional open surgeries, but the expansion of the procedure depends on the experienced surgical team in hepatobiliary surgery, laparoscopic skills, and specialized centers with advanced laparoscopic surgery. Nevertheless, laparoscopic anatomical hemihepatectomies (LAH) are very challenging and technically demanding procedures. Notably, for deep-seated or invisible lesions, the development of LAH is greatly limited because of their deep anatomical position, surgical complication during exposure of the resection plane, and complexity in identifying the boundary of hemihepatectomy, and difficult hemorrhage control [1, 2]. In LAH, identification and anatomical separation of the Glissonean pedicle (GP) at the hepatic hilum and exposure of the landmark hepatic vein represents the critical steps in deciding the transection plane. However, there is a lack of consensus on the standardized approach to LAH [3].

Laennec’s capsule, the liver’s intrinsic membrane, represents an essential structure for the comprehensive understanding of the surgical anatomy of the liver and standardization of the surgical approach to LAH [4, 5]. Some studies have suggested that there is a gap between the extrahepatic GP and Laennec’s capsule that could be used as an anatomical gap to isolate GP and hepatic vein [6–8]. However, LAH based on Laennec’s capsule is rarely reported. Here we described the relevant application of Laennec’s approach for LAH based on Laennec’s capsule and highlighted the surgical anatomical description of the liver and related clinical experience. After that, we retrospectively analyzed the technical details and the surgical outcomes of our standardized approach performed on 15 patients who underwent LAH in our hospital between May 2017 and July 2020. We also investigated the safety and efficacy of this approach to expand our understanding of the membranous anatomy of the liver.

## Materials and methods

### General information

This study comprised 15 patients with benign or malignant neoplasms or hepatolithiasis who underwent LAH between May 2017 and July 2020. Of the 15 patients, 4 patients were diagnosed with hepatic hemangioma, 2 patients had hepatolithiasis, and 9 patients had primary liver cancer. The mean patient age was 62.1 ± 6.5 years, and four were male. The preoperative liver function of patients was Child-Pugh class A, and the indocyanine green retention rate at 15 min (ICG R15) was less than 10%. There was no apparent surgical contraindication before the surgery. None of the lesions affected the anatomy of the first or the second porta hepatis. Laennec’s approach for LAH was performed during the hemihepatectomy by isolating the GP and hepatic vein. The liver tissue specimens adjacent to the GP, hepatic veins, and inferior vena cava (IVC) were collected for hematoxylin and eosin (H&E) and Mallory’s phosphotungstic acid hematoxylin-eosin staining. The study protocol was approved by the Research Ethics Committee of the First Affiliated Hospital of Kangda College of Nanjing Medical University (Approval number: KY20170513001). This study was performed in accordance with the Declaration of Helsinki, and written informed consent was obtained from each patient before surgery.

### Surgical methods

**Fig. 1:**
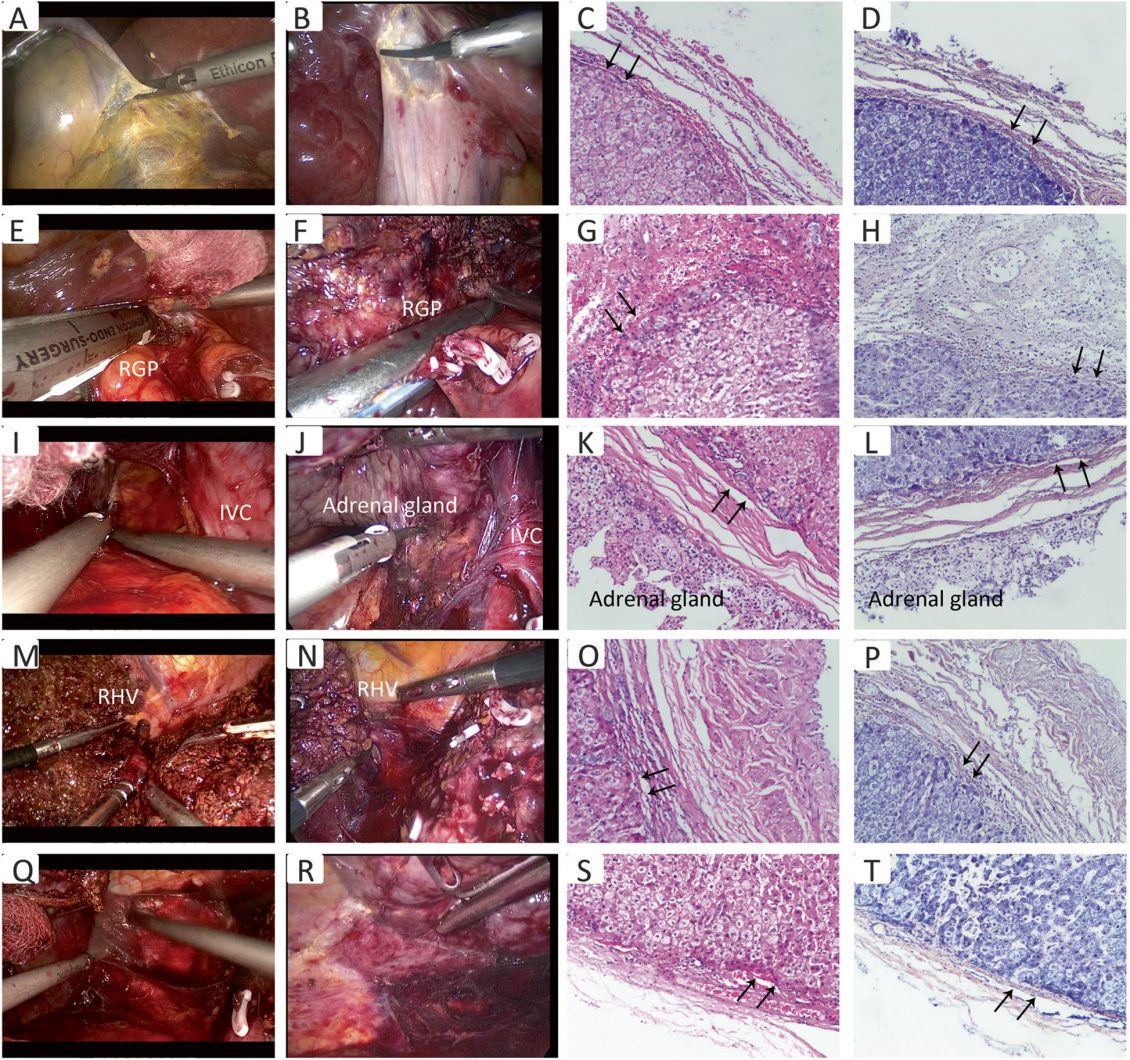
LARH via Laennec’s approach. **A**–**D** Laennec's approach was used to dissect the gallbladder. **E**–**H** The Goldfinger dissector was used to separate the GP of the right liver, and there was a gap between the GP and Laennec's capsule, and endo GIA stapler-cutter was used for the excision of the right liver pedicle. **I**–**L** The ultrasonic scalpel was used to separate the gap between the right liver and the right adrenal gland. **M**–**P** The gap between the right hepatic vein and the liver parenchyma was exposed, and then, endo-GIA stapler-cutter was used for excision. **Q**–**T** The ultrasonic scalpel was used to excise the perihepatic ligament, including the bare area. The arrows point to the Laennec’s capsule and magnified at 100×

All procedures were performed under general anesthesia. Patients were placed in the supine position for resection. The 5-port technique was performed. The pneumoperitoneum pressure was maintained at 14 mmHg. The central venous pressure was maintained between 0 and 3 cm H_2_O during surgery in all cases. The hepatoduodenal ligament was encircled with extraperitoneal blocking tape through Winslow’s foramen by the Pringle maneuver. For right hemihepatectomy, the following procedures were performed: (1) Laennec’s approach for dissection of GP: the hepatic hilar plate was lowered after cholecystectomy. The peritoneum between segment 4 and the surface of GP was incised through Laennec’s capsule. After sufficient dissection, the right GP was ligated using a Goldfinger dissector for traction and transected with a laparoscopic vascular stapler (if the right GP was difficult to dissect, the liver parenchyma dissecting-first method through the hepatic Cantlie line was applied to reveal the root of the right GP [9]). After transection of the right GP, the demarcation line was determined and marked with an electrocoagulation hook (Fig. 1A–H). (2) Laennec’s approach for isolation of hepatic vein: the branches of the middle hepatic vein were dissected under the laparoscopic magnified caudal view, clamped by Hem-o-lok clips, and transected separately. The gap between the main trunk of the right hepatic vein and the Laennec’s capsule was exposed using an ultrasound scalpel to reveal the second porta hepatis. The ligament of IVC and the root of the right hepatic vein were excised individually (Fig. 1I–P). (3) Laennec’s approach for the division of perihepatic tissues: through the anatomical space close to the Laennec’s capsule, the right adrenal gland, IVC and short hepatic veins, and diaphragm were dissected separately. Hemostasis of the bleeding points on the hepatic resection surface or the trunks of hepatic veins was achieved using electrocoagulation or prolene sutures (Fig. 1Q–T).

**Fig. 2:**
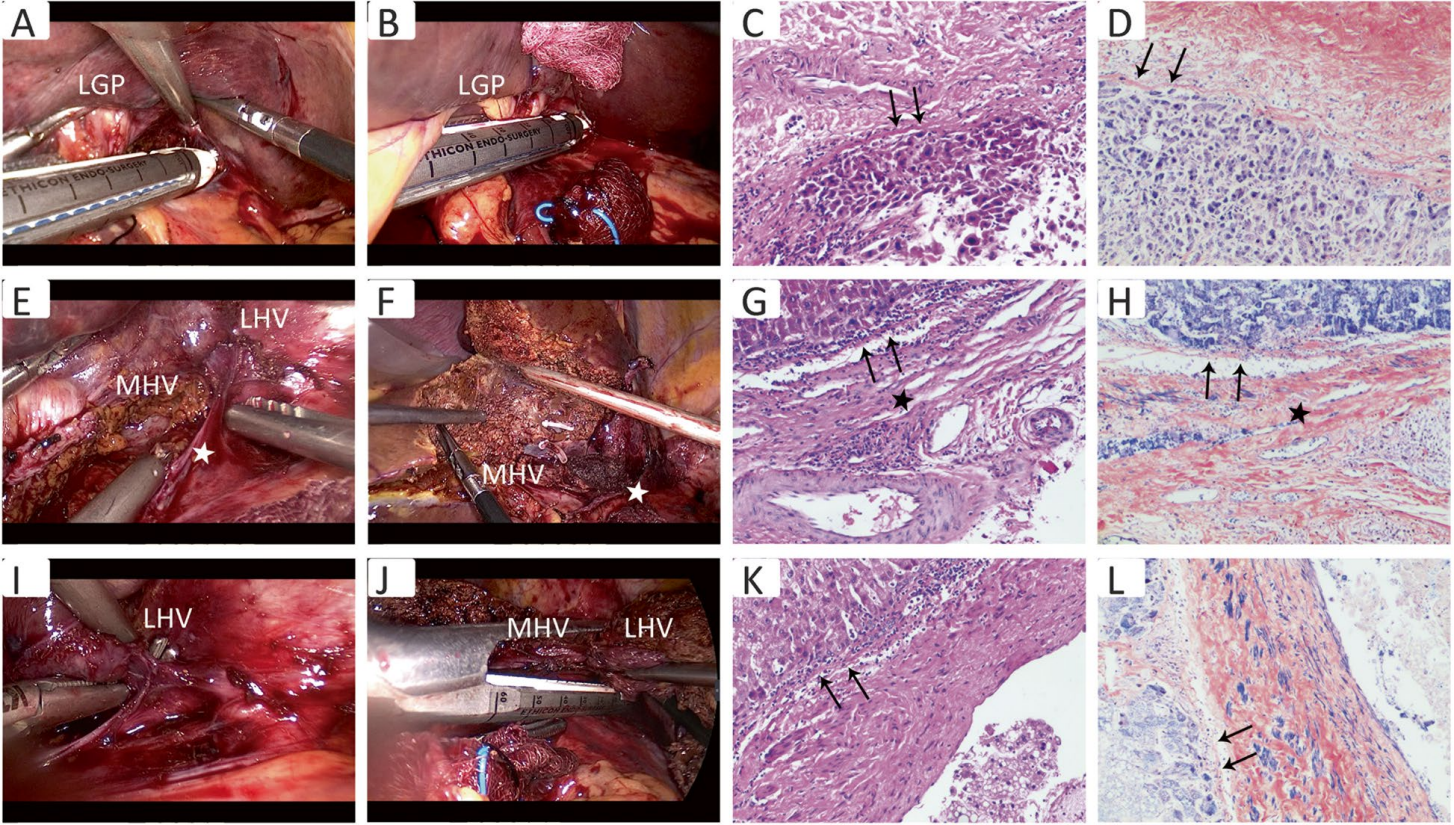
The procedure of LALH through the Laennec’s approach. **A**–**D** The left hepatic GP was isolated from the Laennec’s capsule. When the left hepatic pedicle was dissociated entirely, the ischemic line on the liver surface was marked. **E**–**H** After full dissection of the left GP in front of the Arantius ligament, the middle hepatic vein was then identified. **I**–**L** The root of the left hepatic vein was revealed with the use of the inter-Laennec approach after parenchymal dissection. The arrows point to the Laennec’s capsule, and all staining images were under a microscope (×100). Asterisks point to the Arantius ligament

The procedure followed for the left hemihepatectomy was similar to that of the right hemihepatectomy as described above: (1) Laennec’s approach for separation of the left hepatic pedicle: the gap between the left hepatic pedicle and Laennec’s capsule in front of the Arantius ligament were dissociated, the left hepatic pedicle was dissected after full dissociation, and the ischemic line was marked (Fig. 2A–H). (2) Laennec’s approach for separation of the hepatic vein: the hepatogastric ligament adjacent to the liver was excised, and the gap between the Arantius ligament and the Laennec's capsule was dissected to reveal the gap between the left hepatic vein and the Laennec’s capsule. Left hemihepatectomy was performed by exposing the space between middle hepatic vein and Laennec’s capsule through a dorsal approach (Fig. 2I–L).

### H&E and Mallory staining

Two semi-liver specimens were collected from the livers of two patients undergoing laparoscopic anatomical right and left hemihepatectomies. The specimens were routinely stained with the H&E method. The tissues were fixed in neutral-buffered formalin, embedded in paraffin, and sectioned. The paraffin sections were oxidized with prepared PTAH oxidizer (Mallory staining) for 5 min, bleached with the oxalic acid solution for 1 min, rinsed with tap water for 2 min, washed with distilled water once, and stained with PTAH staining solution for 24–48 h. Then, the sections were sealed after the removal of excess staining with 95% alcohol.

### Postoperative observation and treatment

For the patients’ perioperative care, we applied enhanced recovery after surgery strategy with preventive anti-inflammatory, fluid replacement, and symptomatic support treatment. The vital signs and thoracic and abdominal parameters were closely monitored after surgery, and the abdominal drainage fluid was observed.

### Statistics

Statistical analyses were performed using SPSS 23 statistical software (SPSS Inc., Chicago, IL, USA). Data were expressed as mean and ranges for continuous data, and as numbers with percentages for categorical data. Differences in continuous variables between groups were tested using the Student *t* test. The chi-square or Fisher’s exact test was used to compare categorical variables. *P* <0.05 denotes the presence of statistically significant differences.

## Results

### Dissection of the liver with Laennec’s approach for laparoscopic view

Although the Glissonean approach is widely accepted for hepatectomy, there are yet no anatomical guidelines for the GPs or the hepatic veins isolation. According to the pathological examination, a natural gap exists between the whole parenchyma and adjacent tissues, such as the GPs, the naked area, the hepatic veins, the adrenal gland, and IVC. Based on the anatomical understanding of Laennec’s capsule, Laennec’s approach for LAH was applied as described above. Briefly, the liver mobilization was performed from central location to peripheral isolation with Laennec’s approach for LAH. There are two advantages of this method. The possibility of tumor dissemination caused by squeezing is avoided consistently with the no-touch isolation technique; in situ hemihepatectomy without dissecting the perihepatic ligament decreases liver mobilization difficulty under laparoscopy.

### Outcomes of the patients undergoing LAH with Laennec’s approach

The perioperative characteristics of patients are listed in Table 1. Of the 15 patients, 4 patients were diagnosed with hepatic hemangioma, 2 patients had hepatolithiasis, and 9 patients had hepatocellular carcinoma. The mean patient age was 62.1 ± 6.5 years, and four were male. All patients were successfully treated by surgery, and none of the patients was converted to laparotomy. The operation time was 193 ± 49 min, and the mean blood loss was 247 ± 120 mL. The intraoperative blood transfusion was not needed in any patient. The gastrointestinal decompression tube was not placed during the surgery, and the liquid diet was provided on the first day post-surgery, and the patient was assisted out of bed on the second to third day after surgery. None of the 15 patients had bile leakage and bleeding. According to the Clavien-Dindo classification, there were no or very minimal postoperative complications in 14 patients (grade I, 4). One patient underwent right thoracic puncture and drainage because of dyspnea (grade IIIa, 1), and there was no perioperative mortality. The mean length of postoperative hospital stay was 8.7 ± 2.0 days. No significant difference was observed in the hospital stay or pleural effusion between LARH and LALH (Table 1). The postoperative pathological analysis confirmed that 4 patients had hepatic hemangioma, 2 patients had hepatolithiasis, and 9 patients had primary liver cancer.

**Table 1:** Characteristics of patients

Case	Age	Sex	Diagnosis	Procedure	Operative time (min)	Blood loss (ml)	POHS (days)	Complications	Clavien classification
1	64	F	HCC	LARH	300	300	10		
2	60	F	HM	LALH	220	100	10		
3	57	F	HCC	LARH	160	300	7		
4	66	F	HL	LALH	295	400	9	Pleural effusion	Grade I
5	77	F	HCC	LALH	177	200	11	Ascites	Grade I
6	61	F	HM	LALH	206	200	8		
7	57	M	HCC	LALH	155	100	7		
8	55	F	HCC	LALH	181	50	6		
9	63	M	HCC	LALH	152	400	7		
10	51	F	HM	LARH	180	200	13	Pleural effusion	Grade IIIa
11	66	F	HL	LALH	165	300	7		
12	68	M	HM	LALH	139	400	10	Pleural effusion	Grade I
13	61	M	HCC	LALH	158	150	8		
14	68	F	HCC	LALH	217	200	7		
15	57	F	HCC	LARH	185	400	11	Ascites	Grade I

*Abbreviations: F* female, *M* male, *HCC* hepatocellular carcinoma, *HM* hepatic hemangioma, *HL* hepatolithiasis, *LARH* laparoscopic anatomical right hemihepatectomy, *LALH* laparoscopic anatomical left hemihepatectomy, *POHS* postoperative hospital stay

### Laennec’s capsule does exist around the peripheral hepatic vein

**Fig. 3:**
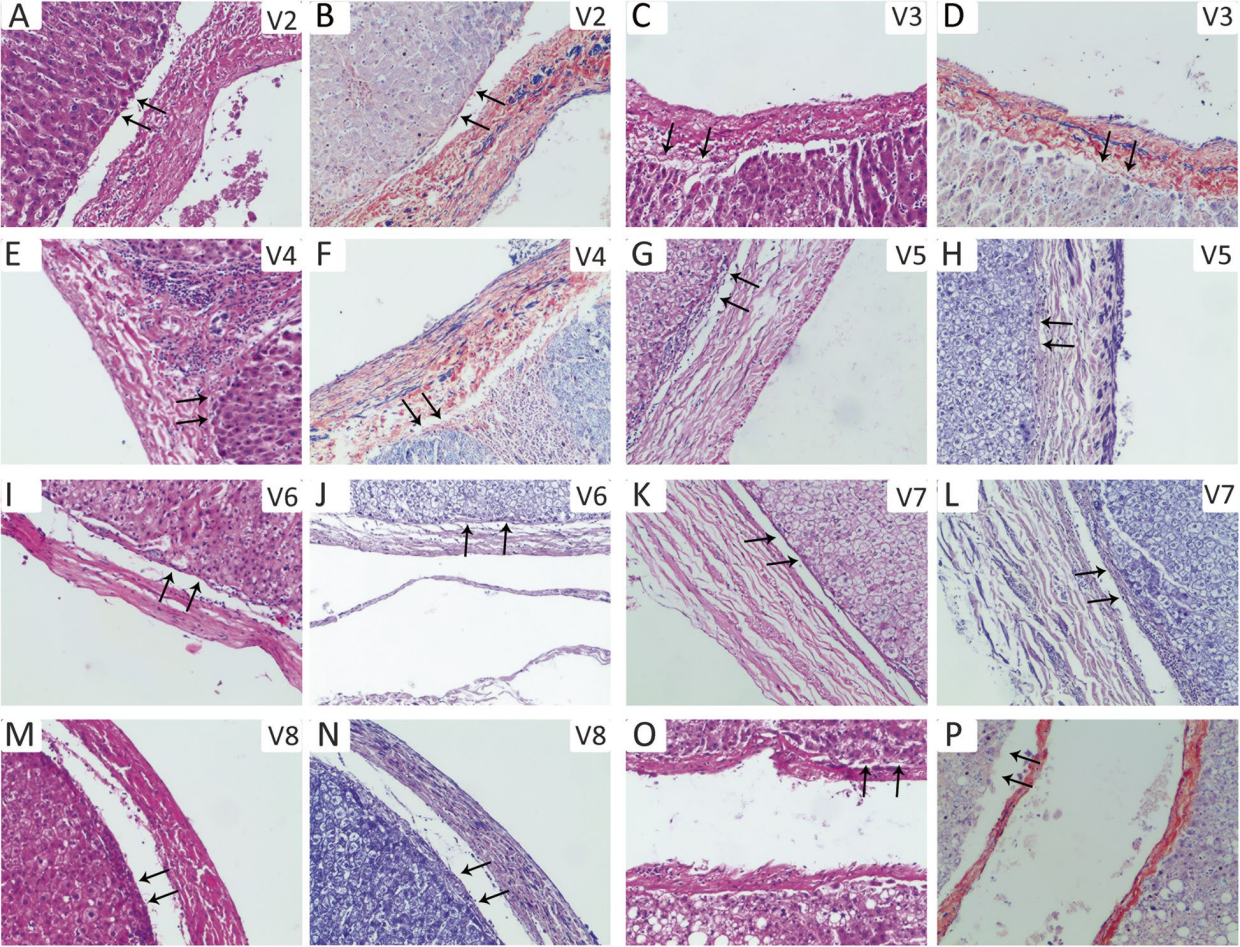
The Laennec’s capsule does exist around the peripheral hepatic veins. The Laennec’s capsule (arrows) was identified to cover the peripheral hepatic vein branch walls of the segment II (**A**, **B**), III (**C**, **D**), IV (**E**, **F**), V (**G**, **H**), VI (**I**, **J**), VII (**K**, **L**), and VII (**M**, **N**) using H&E and Mallory staining. Near the terminal course of the peripheral vein branches, the Laennec’s capsule gradually becomes less apparent or even disappears as the hepatic veins become thinner (**O** and **P**)

At present, it is still controversial whether there is Laennec’s capsule around the branches of the peripheral hepatic vein in the hepatic parenchyma [5, 10–12]. According to the HE and Mallory staining, we not only found that the Laennec’s capsule covers the trunk of the hepatic vein, but also confirmed that there is a gap between the Laennec’s capsule and the peripheral vein branches of the segments II–VII in the hepatic parenchyma (Fig. 3A–N). However, we then observed that near the terminal course of the peripheral vein branches, Laennec’s capsule gradually becomes less apparent or even disappears as the hepatic veins become thinner (Fig. 3O and P). Therefore, the hepatic vein could be dissected along this gap to achieve accurate anatomical segmental hepatectomy from a novel surgical anatomy.

## Discussion

In 1802, French physician Laennec first described the Laennec’s capsule, a proper membrane of the liver, as the distinct structure from the serosa and covered the entire liver. However, Laennec’s capsule was confused with the liver serosa or misunderstood as Glissonean sheath and ignored by liver surgeons for about 200 years. In 2008, Hayashi et al. revealed that the sheath of GPs and hepatic veins is continuous with the liver capsule [4]. Furthermore, in 2017, Sugioka et al. confirmed that Laennec’s capsule not only covers the entire surface of the liver under the serosa but also has gaps surrounding the liver plate, GPs, hepatic veins, and IVC and pronounced that Laennec’s capsule could be applied as an anatomical structure to isolate and expose the GPs [5].

The Glissonean approach is an important technique in open and laparoscopic anatomical hepatectomy [13–15]. Compared to the conventional individual hilar dissection, it produces less intraoperative bleeding and requires less surgery time for transection of the GPs during parenchymal transection. However, while it stresses inflow system control, it has paid little attention to the approach of outflow control or the anatomy of the peri-Glissonean approach.

Laennec’s approach combines the inflow occlusion (Glissonean sheath) and outflow control (hepatic veins) for anatomic hepatectomy. Laennec’s approach serves to isolate GPs and Laennec’s capsule and achieves parenchymal dissection from the trunk of hepatic veins, reaching the goal of anatomical hepatectomy. Laennec’s approach can achieve a bloodless condition in the half liver to be removed while maintaining normal blood flow in the remaining liver tissues, thus reducing the time and frequency of complete blocking of the first hepatic hilum. Meanwhile, there are two advantages of the liver mobilization via Laennec’s approach from the central location to peripheral isolation for LAH. The possibility of tumor dissemination caused by squeezing is avoided consistently with the no-touch isolation technique; in situ hemihepatectomy without dissecting the perihepatic ligament decreases liver mobilization difficulty under laparoscopy. Therefore, Laennec’s approach further ensures the safety of the procedure.

The results of this study revealed that anatomic hepatectomy based on Laennec’s capsule was successfully performed on 15 patients. The mean surgery time was 193 ± 49 min, and the mean loss of blood was 247 ± 120 mL. There are no evident complications or mortality that occurred. Together, these findings suggested that anatomic hepatectomy under laparoscopy based on Laennec’s capsule is a safe, feasible, and effective approach.

The following insights can be drawn from the exploration and practice of anatomic hepatectomy based on Laennec’s capsule under laparoscopy: (1) Before the procedure, an improved understanding of the anatomical position of GPs and the presence (or absence) of anatomical variation based on liver enhanced CT and MRCP examination helps to isolate the GPs during surgery. (2) During surgery, the correct operating plane must be identified. Laennec’s capsule is close to the entire liver, so anatomical separation should be performed in Laennec’s inter-membrane space when dissecting the first, second, and third hepatic hilum, and perihepatic tissues. The operation may not enter the sneath or go deep into the liver parenchyma. (3) In case of the thickening and fattening of the middle lobe of the liver, or the trapping of the first hepatic hilum in the liver parenchyma due to liver atrophy, hepatic hilum translocation, or other reasons, it may be very difficult to separate Laennec’s capsule from GPs under laparoscopy. In that case, no separation should be forced, as it may cause unnecessary medical injuries. We can first transect the liver parenchyma along the anatomy Cantlie line and then excise when the root of GPs to be removed is revealed. (4) In-place excision of semi-liver excision should be performed from central to peripheral isolation, thus avoiding the difficulty of mobilizing the liver and removing the peripheral ligaments of the liver under laparoscopy. (5) The gap of the post-hepatic IVC can be revealed together with the trunk of hepatic veins, so as to accurately show the liver section. There are also limitations to the application of Laennec’s capsule for laparoscopic anatomic hepatectomy. One of the contraindications of this procedure is that the tumor location is adjacent to, or infringes upon GPs, or the trunk of hepatic veins. Therefore, it is important to ensure that the tumor is separated by a safe distance from GPs or the hepatic venous trunk.

At present, some scholars believe that there exists no Laennec’s capsule around peripheral branches of the hepatic veins [5, 10, 12]. However, we confirmed through H&E and Mallory staining that Laennec’s capsule also covers the branches of the peripheral hepatic vein of the segments II–VIII, and there is a gap between Laennec’s capsule and the veins. In precision liver excision, dissecting Laennec’s capsule gap along the liver venous trunk and its branches can effectively achieve segmental anatomic liver excision.

This study highlights that Laennec’s approach provides a new perspective for laparoscopic anatomical hepatectomy. It is beneficial to the procedure and standardization of laparoscopic anatomical hepatectomy. Laennec’s capsule can be used as an anatomical marker for anatomical hepatectomy, expecting to promote liver surgeons’ understanding of liver membrane anatomy and the development of LAH.

## Conclusions

Laennec’s approach is safe and feasible for LAH. Precise isolation of Laennec’s approach based on Laennec’s capsule helps to standardize the surgical techniques for laparoscopic anatomical hepatectomy.

## Data Availability

Please contact the corresponding author with request for data.

## Abbreviations

LARH: Laparoscopic anatomical right hemihepatectomy
LALH: Laparoscopic anatomical left hemihepatectomy
LAH: Laparoscopic anatomical hemihepatectomy
GP: Glissonean pedicle
IVC: Inferior vena cava
ICGR15: Indocyanine green retention rate at 15 min

## References

[1] Kim JH, Choi JW (2019). Intrahepatic Glissonian approach to the ventral aspect of the Arantius ligament in laparoscopic left hemihepatectomy. World J Surg..

[2] Kim JH, Kim H (2021). Laparoscopic right hemihepatectomy using the Glissonean approach: detachment of the hilar plate (with video). Ann Surg Oncol..

[3] Wakabayashi G, Cherqui D, Geller DA, Buell JF, Kaneko H, Han HS (2015). Recommendations for laparoscopic liver resection: a report from the second international consensus conference held in Morioka. Ann Surg..

[4] Hayashi S, Murakami G, Ohtsuka A, Itoh M, Nakano T, Fukuzawa Y (2008). Connective tissue configuration in the human liver hilar region with special reference to the liver capsule and vascular sheath. J Hepatobiliary Pancreat Surg..

[5] Sugioka A, Kato Y, Tanahashi Y (2017). Systematic extrahepatic Glissonean pedicle isolation for anatomical liver resection based on Laennec’s capsule: proposal of a novel comprehensive surgical anatomy of the liver. J Hepatobiliary Pancreat Sci..

[6] Kiguchi G, Sugioka A, Kato Y, Uyama I (2019). Laparoscopic S7 Segmentectomy using the inter-Laennec approach for hepatocellular carcinoma near the right hepatic vein. Surg Oncol..

[7] Monden K, Sadamori H, Hioki M, Sugioka A (2020). Laparoscopic anatomic segmentectomy 8 using the outer-Laennec approach. Surg Oncol..

[8] Hu Y, Shi J, Wang S, Zhang W, Sun X, Sun B (2019). Laennec’s approach for laparoscopic anatomic hepatectomy based on Laennec’s capsule. BMC Gastroenterol..

[9] Xiao L, Wang Z, Zhou L (2020). “Liver parenchyma dissecting-first” method facilitates the Glissonean pedicle approach in anatomical laparoscopic hepatolobectomy. Ann Transl Med..

[10] Shirata C, Hasegawa K, Halkic N, Kokudo N (2019). Laennec’s capsule does not exist around the peripheral hepatic veins. J Hepatobiliary Pancreat Sci..

[11] Sugioka A (2019). Re: Laennec’s capsule does not exist around the peripheral hepatic veins. J Hepatobiliary Pancreat Sci..

[12] Shirata C, Kokudo T, Gillet M, Uldry E, Demartines N, Kokudo N (2020). Reappraisal of Laennec’s capsule. Surg Oncol..

[13] Ferrero A, Lo TR, Giovanardi F, Langella S, Forchino F, Russolillo N (2021). Laparoscopic right posterior anatomic liver resections with Glissonean pedicle-first and venous craniocaudal approach. Surg Endosc..

[14] Lee JH, Han DH, Jang DS, Choi GH, Choi JS (2016). Robotic extrahepatic Glissonean pedicle approach for anatomic liver resection in the right liver: techniques and perioperative outcomes. Surg Endosc..

[15] Yamamoto M, Ariizumi SI (2018). Glissonean pedicle approach in liver surgery. Ann Gastroenterol Surg..

